# Supplementary material for: Multi‐dataset identification of innovative feature genes and molecular mechanisms in keratoconus
Source: J Cell Mol Med. 2024 Sep 19;28(18):e70079. doi: 10.1111/jcmm.70079 (PMC11412914; doi:10.1111/jcmm.70079)
Supplement: Supplementary file 2 — Material S2. [file JCMM-28-e70079-s002.zip › 7.GSEA analysis/2.ATOH7/KEGG_NITROGEN_METABOLISM.html]

Details for gene set KEGG\_NITROGEN\_METABOLISM[GSEA]

|  || Dataset | ATOH7.group.cls#h\_versus\_l |
| Phenotype | group.cls#h\_versus\_l |
| Upregulated in class | h |
| GeneSet | KEGG\_NITROGEN\_METABOLISM |
| Enrichment Score (ES) | 0.6005479 |
| Normalized Enrichment Score (NES) | 1.5824646 |
| Nominal p-value | 0.036893204 |
| FDR q-value | 0.28383857 |
| FWER p-Value | 0.638 |
Table: GSEA Results Summary

  

Fig 1: Enrichment plot: KEGG\_NITROGEN\_METABOLISM      
 Profile of the Running ES Score & Positions of GeneSet Members on the Rank Ordered List

  

| SYMBOL | TITLE | RANK IN GENE LIST | RANK METRIC SCORE | RUNNING ES | CORE ENRICHMENT || 1 | CA12 | na | 93 | 0.778 | 0.1864 | Yes |
| 2 | CA2 | na | 1268 | 0.412 | 0.2527 | Yes |
| 3 | CA8 | na | 1609 | 0.376 | 0.3343 | Yes |
| 4 | CA9 | na | 2182 | 0.331 | 0.3982 | Yes |
| 5 | CA1 | na | 3216 | 0.269 | 0.4338 | Yes |
| 6 | CA14 | na | 3450 | 0.258 | 0.4897 | Yes |
| 7 | HAL | na | 4458 | 0.219 | 0.5141 | Yes |
| 8 | CTH | na | 5078 | 0.199 | 0.5445 | Yes |
| 9 | CPS1 | na | 5543 | 0.184 | 0.5759 | Yes |
| 10 | CA4 | na | 6349 | 0.161 | 0.5918 | Yes |
| 11 | GLS2 | na | 7210 | 0.138 | 0.6005 | Yes |
| 12 | CA6 | na | 8367 | 0.111 | 0.5942 | No |
| 13 | AMT | na | 11022 | 0.066 | 0.5337 | No |
| 14 | CA7 | na | 13372 | 0.037 | 0.4749 | No |
| 15 | GLUD2 | na | 13502 | 0.035 | 0.4798 | No |
| 16 | ASNS | na | 13578 | 0.035 | 0.4860 | No |
| 17 | GLS | na | 16616 | 0.008 | 0.4004 | No |
| 18 | GLUL | na | 16859 | 0.006 | 0.3949 | No |
| 19 | CA3 | na | 19144 | -0.014 | 0.3325 | No |
| 20 | CA13 | na | 20772 | -0.029 | 0.2928 | No |
| 21 | CA5B | na | 22015 | -0.043 | 0.2675 | No |
| 22 | GLUD1 | na | 22595 | -0.050 | 0.2628 | No |
| 23 | CA5A | na | 32872 | -0.357 | 0.0535 | No |
Table: GSEA details [plain text format]

  

Fig 2: KEGG\_NITROGEN\_METABOLISM      
 Blue-Pink O' Gram in the Space of the Analyzed GeneSet

  

Fig 3: KEGG\_NITROGEN\_METABOLISM: Random ES distribution      
 Gene set null distribution of ES for **KEGG\_NITROGEN\_METABOLISM**
